# Pathogenicity Analysis and Molecular Characterization of Three *Avr* Genes in *Magnaporthe oryzae* Population from Central Jilin Province

**DOI:** 10.3390/microorganisms14051017

**Published:** 2026-04-30

**Authors:** Yimeng Wang, Nuozhou Zhang, Rui Han, Aozheng Lu, Nan Nan, Dayong Li, Wenxian Sun

**Affiliations:** Jilin Provincial Key Laboratory of Green Management of Crop Pests and Diseases, College of Plant Protection, Jilin Agricultural University, Changchun 130118, China; 15567731311@163.com (Y.W.); zhangnuozhou@jlau.edu.cn (N.Z.); hanry0930@163.com (R.H.); 18254746325@163.com (A.L.); 08042@cau.edu.cn (W.S.)

**Keywords:** *Oryza sativa*, *Magnaporthe oryzae*, avirulence gene, pathogenicity, virulence frequency

## Abstract

Rice fungal blast, one of the most devastating diseases caused by *Magnaporthe oryzae*, poses a severe threat to global rice production. For the breeding and deployment of rice varieties with blast resistance, it is critical to elucidate the frequencies and genetic variations in avirulence genes among *M. oryzae* populations. In this study, a total of 294 *M. oryzae* isolates were collected in 2022 from central Jilin Province, China. Pathogenicity assays on 24 monogenic rice lines revealed extensive virulence variations among the 294 isolates, with highly pathogenic strains being dominant and clear geographic differences in pathogenicity profiles. Resistance frequencies differed markedly among 24 monogenic lines, with *Pi3*, *Pit*, *Pi7*, *Pikh*, *Pik*, and *Pia* showing resistance rates over 50% and *Pish* exhibiting the lowest efficacy. Moreover, resistance profiles varied significantly across four sampling regions in central Jilin Province, with *Pit* being the most effective in Changchun and Jilin, *Pi3* in Tonghua, and *Pikm* in Liaoyuan. In addition, the *Avr* genotypes of the isolates were postulated based on phenotypic data from the monogenic rice lines. Among the postulated *Avr* genotypes, the frequencies of *Avr*-*Pi11* and *Avr*-*Pish* were the lowest at 29.25%. Furthermore, molecular characterization of three *Avr* genes (*Avr-Pi9*, *Avr-Pita2*, and *Avr-Pizt*) was performed by sequencing a subsample of 50 randomly selected isolates. Natural mutation sites were identified in *Avr-Pita2* and *Avr-Pizt*, which were located within the coding sequence regions, leading to non-synonymous mutations and nonsense mutations that cause premature termination. Notably, no mutation was detected within the coding sequences of *Avr-Pi9*. Collectively, the findings provide a theoretical basis for breeding blast-resistant rice varieties that can be deployed in central Jilin Province, China.

## 1. Introduction

Rice (*Oryza sativa* L.) ranks among China’s top three cereal crops in both planting area and total yield [1]. Rice blast, caused by the filamentous fungus *Magnaporthe oryzae*, ranks as one of the most devastating diseases in rice [2]. It causes 10–30% yield losses worldwide and poses a substantial threat to global rice production and food security [3,4]. This highlights the urgent need for rice blast prevention and management strategies to safeguard sufficient food production. Although seed disinfection and fungicide application can inhibit rice blast outbreaks, the most effective and economical approach to control this disease is to breed highly resistant rice cultivars [5,6]. Therefore, elucidating the molecular interaction between rice and *M. oryzae* provides a fundamental basis for the rational control of rice blast and the development of novel resistant rice varieties [7]. Plants and pathogens have long maintained an antagonistic relationship, engaged in an ongoing evolutionary arms race to secure their respective survival—a dynamic that will persist as long as both coexist on Earth. To gain an advantage over one another, plants and their pathogens have evolved elaborate counterstrategies [8]. On the one hand, plants deploy a variety of surveillance molecules, such as pattern recognition receptors (PRRs), to continuously detect conserved pathogen signatures collectively referred to as pathogen-associated molecular patterns (PAMPs) [9]. On the other hand, plant resistance (*R*) genes and pathogen avirulence (*Avr*) genes act as key molecular determinants in this interaction [10]. According to the gene-for-gene hypothesis, an avirulence gene product of a pathogen is specifically recognized by the cognate resistance gene product in host cells, thus triggering robust defenses against pathogen infection [11].

Numerous researchers are devoted to the identification of *R* genes for breeding disease-resistant rice cultivars. So far, approximately 500 blast resistance loci have been genetically mapped, and over 62 *R* genes have been successfully isolated and cloned, including *Pib* [12], *pi21* [13], *Pizt* [14], *Pi2* [14], *Pia* [15], *Pi64* [16], *Pikh* [17], and others [3,18]. In addition, multiple rice *R* genes have been reported to harbor multiple alleles, such as *Pik*, *Pikm*, *Pikp*, *Piks*, and *Pikh*, which confer resistance against distinct races of *M. oryzae* [19]. Correspondingly, 12 *Avr* genes have been successfully cloned, namely, *PWL1* [20], *PWL2* [21], *Avr1-CO39* [22], *ACE1* [23], *Avr-Pi54* [24], *Avr-Pita* [25], *Avr-Pizt* [26], *Avr-Pia* [27], *Avr-Pii* [27], *Avr-Pik/km/kp* [27], *Avr-Pib* [28], and *Avr-Pi9* [29], each of which is specifically recognized by its cognate *R* gene [3]. *M. oryzae Avr* genes are highly variable and often experience deletions, translocations, transposon insertions, and base substitutions [6,30,31,32,33]. A persistent evolutionary conflict exists between rice and *M. oryzae*. The rapid adaptive evolution of *M. oryzae* populations enables emerging virulent strains to overcome *R*-gene-mediated resistance soon after resistant cultivars are released [30,34]. Therefore, deployment of blast resistance cultivars requires monitoring of the dynamics of *M. oryzae* races and gaining information on the current frequency of *Avr* genes in *M. oryzae* populations.

In this study, 294 field isolates of *M. oryzae* were collected from central Jilin Province, an epidemic region of rice blast. We evaluated the pathogenicity of these isolates using 24 monogenic rice lines and further inferred *Avr* profiles based on inoculation phenotypes [35,36]. The findings are anticipated to provide a valuable theoretical reference for the development and rational deployment of blast-resistant rice varieties in this region.

## 2. Materials and Methods

### 2.1. Fungal Isolates and Culture

*M. oryzae* isolates were collected as described previously [37]. Briefly, *M. oryzae*-infected leaves of the susceptible variety Lijiangxintuanheigu (LTH) were collected from four hotspots of rice blast disease in central Jilin Province in 2022. In each region, 2–3 representative fields were selected, with the following sampling locations: Changchun city (CC, including suburban areas, Jiutai, and Shuangyang); Jilin city (JL, including Yongji, Panshi, and Huadian); Tonghua city (TH, including Huinan, Meihekou, and Liuhe); and Liaoyuan city (LY, including Dongfeng and Dongliao). Single spores were isolated from individual disease lesions with a sterilized needle under a microscope, cultured on oatmeal–tomato agar (OTA) plates (50 g/L oatmeal, 200 mL/L tomato juice, and 15 g/L agar), and finally stored on sterilized filter paper at −20 °C.

### 2.2. Rice Materials

All rice MLs developed by the International Rice Research Institute (IRRI) carry a single major blast *R* gene in the LTH genetic background [36]. Using LTH as a negative control, 24 MLs carrying the *R* genes *Pia*, *Pii*, *Piks*, *Pik*, *Pikp*, *Pikh*, *Piz*, *Piz5*, *Pizt*, *Pi-ta*, *Pib*, *Pit*, *Pish*, *Pi1*, *Pi3*, *Pi5*, *Pi7*, *Pi9*, *Pi12*, *Pi19*, *Pikm*, *Pi20*, *Pi-ta2*, and *Pi11* were used for pathogenicity assays and resistance spectrum analysis (Table S1). The seedlings were grown to the three- to four-leaf stage for inoculation assays in a greenhouse.

### 2.3. Pathogenicity Assay

*M. oryzae* isolates were cultured on OTA medium plates at 28 °C for 7 days. The mycelia were scraped with sterilized cotton swabs, and the plates were subsequently exposed to continuous light at 28 °C for an additional 3 to 5 days to promote sporulation. The conidia were washed with 0.025% Tween-20 and were then filtered with six layers of cheesecloth. Conidiospore concentrations were determined using a hemocytometer and adjusted to 1 × 10^5^ conidia/mL for inoculation.

Wound inoculation was performed as previously reported [38]. Briefly, the second youngest leaves were detached and cut into 5–7 cm fragments, which were then placed onto a 90 mm Petri dish with wet filter paper. The leaf fragments were scratched along the main vein using a pin. Three incisions approximately 2–3 mm long were made per leaf segment, with care taken not to pierce through the leaf. Subsequently, 10 μL of conidium suspension was drop-inoculated at the wound sites. Three inoculated fragments were sealed in a Petri dish and incubated in dark at 26 °C with 90% relative humidity for 24 h. Lesion size was assessed at 4 to 7 days post-inoculation to evaluate the pathogenicity of isolates [38].

**Figure 1:**
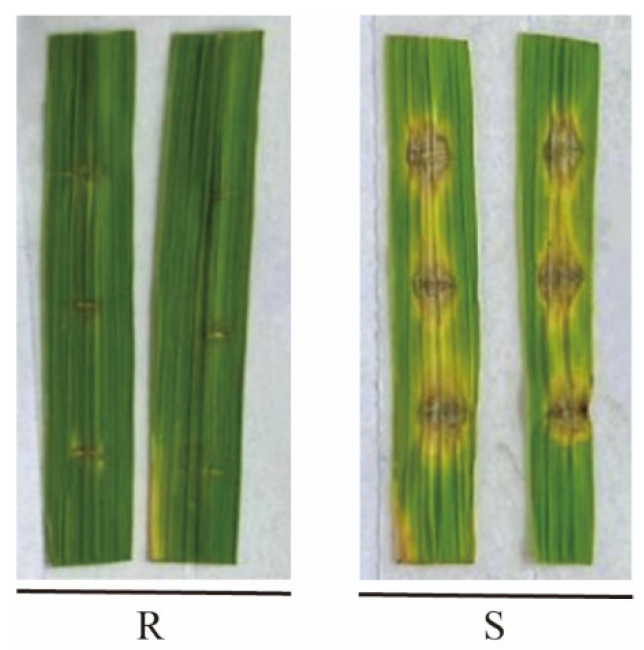
Reaction types of isolates on MLs: resistance (R) and susceptible (S).

The reaction types were categorized into two groups: incompatible (R) and compatible (S) reactions (Figure 1). In the incompatible reaction, lesions appeared dark brown and were restricted to the wounded area without a distinctive center, with a diameter of less than 5 mm. In the compatible reaction, lesions were spindle-shaped, featuring gray centers, brown margins, and yellow halos, and their diameter exceeded 5 mm [38].

### 2.4. Identification of Avr Genes

In inoculation assays, if a tested isolate induced an incompatible reaction on a monogenic line harboring a single major *R* gene, we inferred that the isolate harbored the corresponding *Avr* gene; conversely, a compatible reaction indicated the absence of this *Avr* gene. Based on this principle, the *Avr* gene profiles of all isolates were deduced based on the resistant or susceptible phenotypes of the isolates against the 24 monogenic lines.

The pathogenic frequency (PF) of each isolate was defined as follows: PF (%) = (number of monogenic lines susceptible to the isolate/total number of monogenic lines) × 100. Isolates were further categorized by the pathogenicity level using the PF criterion: PF ≥ 70% indicated extremely high pathogenicity (EHP); 70% > PF ≥ 50% indicated high pathogenicity (HP); 50% > PF ≥ 20% indicated moderate pathogenicity (MP); and PF < 20% indicated low pathogenicity (LP) [39].

In addition, the resistance frequency (RF) of each ML was calculated using the following formula: RF (%) = (number of isolates with incompatible reaction to monogenic lines/total number of isolates) × 100 [40].

### 2.5. DNA Extraction, Avr Gene Detection, and Characterization of Mutations

For fungal genomic DNA extraction, purified fungus isolates preserved on filter paper were cultured on oatmeal agar for 5–7 days. Mycelial plugs of 0.5 cm diameter were then inoculated into 50 mL liquid complete medium (CM) and incubated in darkness at 28 °C with shaking at 160 rpm for 3 days. Mycelia were harvested by gauze filtration, blotted dry with filter paper, and ground into powder under liquid nitrogen. Genomic DNA was extracted with a Fungi Genomic DNA Extraction Kit (Solarbio Science & Technology, Beijing, China) following the manufacturer’s instructions. Primers for the detection of *Avr* genes were designed based on the published sequences of *Avr-Pizt*, *Avr-Pita2*, and *Avr-Pi9* (Table S2). A 50 μL volume of the PCR mixture consisted of 25 μL of 2 × Phanta Max buffer, 1 μL of 10 mM dNTP mix, 1 μL of Phanta Max super-fidelity DNA polymerase (all from Vazyme Biotech Co., Ltd., Nanjing, China), 1 μL each of 10 μM primers, approximately 500 ng of fungal genomic DNA, and ddH_2_O. The PCR reaction conditions were: pre-denaturation at 95 °C for 3 min; 35 cycles of denaturation at 95 °C for 15 s, annealing at 58 °C for 15 s, and extension at 72 °C for 30 s; final extension at 72 °C for 5 min. The PCR products were sent to Sangon Biotech (Shanghai) Co., Ltd. (Changchun, China) for sequencing.

### 2.6. Statistical Analysis

**Figure 2:**
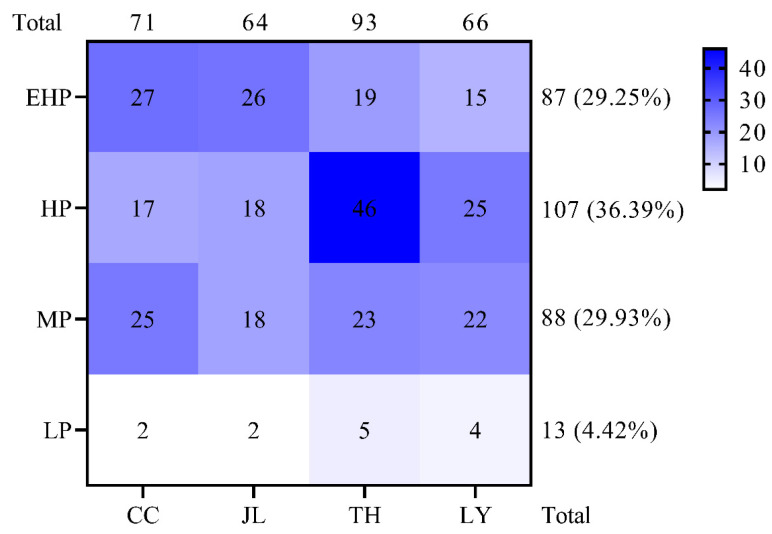
The pathogenicity frequency of 294 *M. oryzae* isolates. Numbers represent the numbers of isolates tested. EHP, extremely high pathogenicity; HP, high pathogenicity; MP, moderate pathogenicity; LP, low pathogenicity; CC, Changchun; JL, Jilin; TH, Tonghua; LY, Liaoyuan.

**Figure 3:**
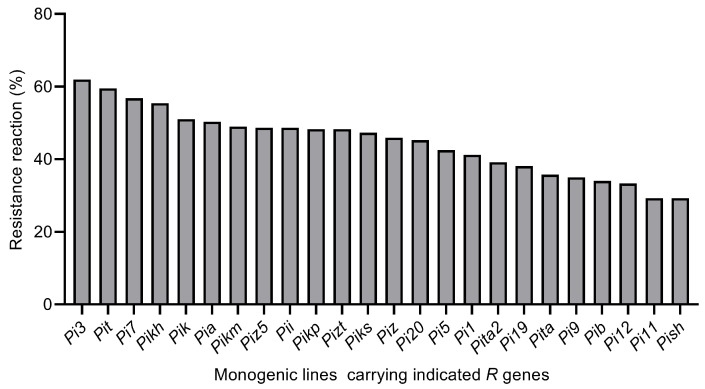
The resistance frequency of MLs to isolates collected from central Jilin Province in 2022.

**Figure 4:**
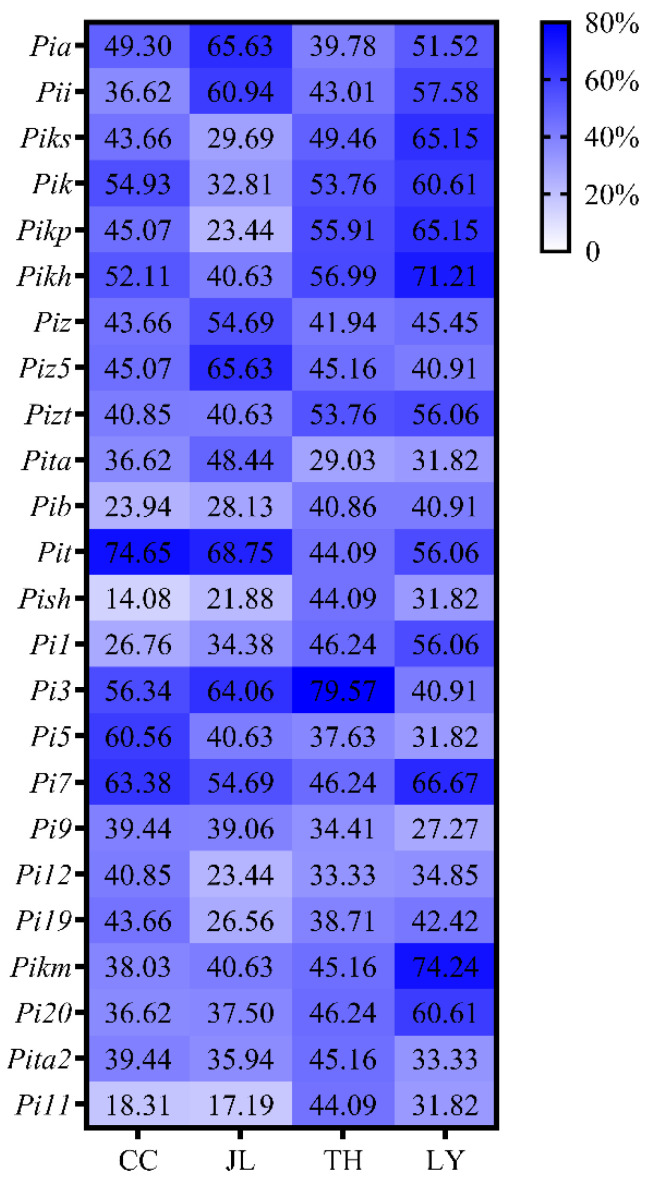
The resistance frequency of MLs to isolates collected from Changchun, Jilin, Tonghua, and Liaoyuan.

All raw data calculation and statistical sorting were performed using Microsoft Excel 2016. The graphical presentation of experimental data in Figure 2, Figure 3 and Figure 4 was generated using GraphPad Prism 9 software.

## 3. Results

### 3.1. Pathotypic Diversity of Rice Blast Isolates Collected from Central Jilin Province

*M. oryzae*-infected leaves of the susceptible variety LTH were collected from four regions of Jilin Province, namely, Changchun city (CC), Jilin city (JL), Tonghua city (TH), and Liaoyuan city (LY), and they were used for single-spore isolation (Figure S1). A total of 294 single-spore isolates were obtained from the infected leaves in 2022, with 71, 64, 93, and 66 isolates from Changchun, Jilin, Tonghua, and Liaoyuan, respectively (Table 1).

Pathogenicity assays demonstrated that the virulence of these isolates to the 24 MLs varied considerably. The susceptible control variety LTH was susceptible to all isolates. The pathogenicity profiles of these isolates on the MLs are presented in Table S3. The frequencies of virulent and avirulent isolates against each of the 24 MLs differed significantly. For example, isolate JL3 induced a susceptible reaction on all 24 lines (Table S3). Among the 294 isolates tested, 106 (36.39%) exhibited high pathogenicity, representing the most dominant pathogenic category. This was followed by 88 isolates (29.93%) with moderate pathogenicity, 87 (29.25%) with extremely high pathogenicity, and 13 (4.42%) with low pathogenicity (Figure 2). Furthermore, isolates with extremely high pathogenicity accounted for the largest proportion in Changchun (36.62%) and Jilin (40.62%), respectively. By contrast, isolates with high pathogenicity were the most dominant in Tonghua and Liaoyuan, accounting for 49.46% and 37.88%, respectively. These findings indicate that the isolates with high pathogenicity constitute the predominant population within the tested collection.

### 3.2. Resistance Analysis of MLs to Rice Blast Isolates Collected from Central Jilin Province

Resistance levels varied substantially among different monogenic rice lines, as each line conferred resistance to a different number of the collected isolates. Among the 294 isolates collected from central Jilin Province, resistance frequencies conferred by *Pi3*, *Pit*, *Pi7*, *Pikh*, *Pik*, and *Pia* exceeded 50%, with *Pi3* showing the highest efficacy at 61.90%. In contrast, the lowest resistance frequency (29.25%) was detected against the monogenic line carrying *Pish* (Figure 3).

Resistance frequencies also differed among isolates from the four sampling regions. Among the 71 isolates from Changchun, the resistance frequencies conferred by *Pit*, *Pi7*, *Pi5*, *Pi3*, *Pik*, and *Pikh* were above 50%, with *Pit* being the most effective at 74.65%; the lowest resistance frequency (14.08%) was detected for the line carrying *Pish* (Figure 4). Among the 64 isolates from Jilin, the resistance frequencies conferred by *Pit*, *Pia*, *Piz5*, *Pi3*, *Pii*, *Pi7*, and *Piz* exceeded 50%, with *Pit* showing the highest efficacy at 68.75%, whereas the lowest resistance frequency (17.19%) was found for *Pish* (Figure 4). For the 93 isolates collected from Tonghua, the resistance frequencies conferred by *Pi3*, *Pikh*, *Pikp*, *Pizt*, and *Pik* were greater than 50%, with *Pi3* exhibiting the highest efficacy at 79.57%; the lowest resistance frequency (29.03%) was recorded for the line carrying *Pi-ta* (Figure 4). Among the 66 isolates collected from Liaoyuan, the resistance frequencies conferred by *Pikm*, *Pikh*, *Pi7*, *Pikp*, *Piks*, *Pik*, *Pi20*, *Pii*, *Pizt*, *Pi1*, *Pit*, and *Pia* surpassed 50%, with *Pikm* being the most effective at 74.24%, while the lowest resistance frequency (27.27%) was observed for *Pi9* (Figure 4). In summary, *Pit* was the most effective gene in Changchun and Jilin, *Pi3* performed the best in Tonghua, and *Pikm* was the most effective in Liaoyuan (Figure 4).

### 3.3. Avr Gene Analysis

Based on the inoculation assays and the gene-for-gene hypothesis, the repertoire of corresponding *Avr* genes carried by each isolate was inferred and summarized in Table S4. The number of *Avr* genes harbored in individual *M. oryzae* isolates varied substantially, ranging from 0 to 22. Isolate JL3 carried no *Avr* genes, whereas isolates TH50 and LY27 harbored 22 *Avr* genes. Notably, among the *M. oryzae* isolates collected from central Jilin Province, the postulated frequencies of *Avr-Pi11* and *Avr-Pish* were the lowest, followed by *Avr-Pi12* and *Avr-Pib* at 33.33% and 34.01%, respectively. These findings indicate that *Avr-Pi11*, *Avr-Pish*, *Avr-Pi12*, and *Avr-Pib* occurred at relatively low frequencies in field isolates from central Jilin Province. Clear regional differences were also observed in *Avr* gene composition. In Changchun, *Avr-Pi11* exhibited the lowest postulated frequency at 18.31%, followed by *Avr-Pib* and *Avr-Pi1*, at 23.94% and 26.76%, respectively. In Jilin, *Avr-Pi11* was also the least inferred frequent *Avr* gene at 17.19%, followed by *Avr-Pish*, *Avr-Pikp*, and *Avr-Pi12* at 21.87%, 23.44%, and 23.44%, respectively. In Tonghua, *Avr-Pita* displayed the lowest inferred frequency at 29.03%, followed by *Avr-Pi12* and *Avr-Pi9* at 33.33% and 34.41%, respectively. In Liaoyuan, *Avr-Pi9* was the lowest postulated frequency (27.27%), followed by *Avr-Pita*, *Avr-Pit*, *Avr-Pi5*, and *Avr-Pi11* at 31.82%. Together, these results reveal distinct geographic differentiation in the *Avr* gene composition of *M. oryzae* populations across central Jilin Province.

### 3.4. Natural Variations in Avr Genes

**Figure 5:**
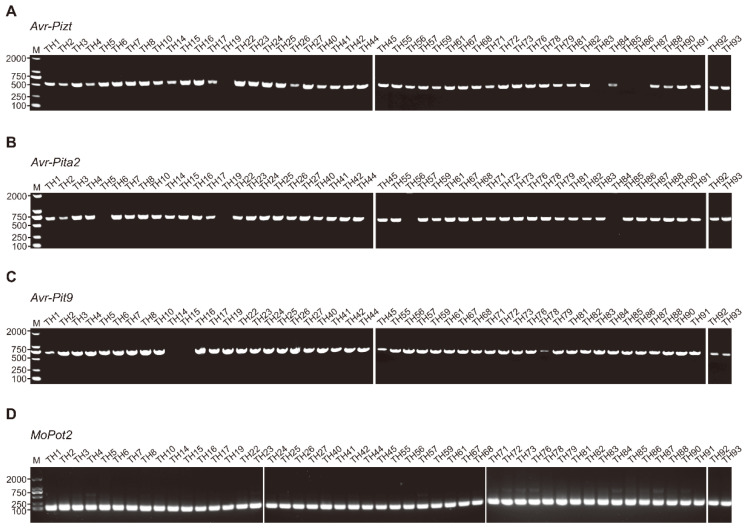
Polymerase chain reaction (PCR) detection of the three *Avr* genes from *M. oryzae* isolates. (**A**) PCR products of *Avr*-*Pizt*. (**B**) PCR products of *Avr*-*Pita2*. (**C**) PCR products of *Avr*-*Pi9*. (**D**) PCR products of *MoPto2* as an internal control. M, marker.

Based on the distribution and abundance of *Avr* genes, the application of *R* genes to enhance rice blast resistance in central Jilin Province exhibits considerable promise. In this study, a total of 50 *M. oryzae* isolates were randomly selected for genomic DNA extraction. The coding sequences (CDSs) of *Avr-Pizt*, *Avr-Pita2*, and *Avr-Pi9* were amplified via PCR and subsequently sequenced. For *Avr-Pizt*, amplicons were successfully obtained from 46 isolates, while four isolates failed to have PCR products (Figure 5A). Among the 46 isolates, 19 were further sequenced due to their observed virulence for the monogenic line K9, which carries the resistance gene *Pizt*. Sequence analysis revealed a G-to-A substitution at the nucleotide position 101 (relative to the ATG start codon) in isolate TH5. This nonsense mutation replaced tryptophan (W) with a stop codon. No sequence variations were detected in the other 18 sequenced isolates (Table S5). For *Avr-Pita2*, PCR products were amplified from 46 isolates, with 4 isolates showing no amplification (Figure 5B). Among these 46 isolates, 21 were selected for sequencing based on their compatible reaction with the monogenic line K23 (harboring *Pi-ta2*). Sequence alignment identified an A-to-G substitution at the nucleotide position 247 in four isolates (TH3, TH10, TH61 and TH73), leading to an amino acid change from aspartic acid (D) to asparagine (N). The remaining 17 isolates displayed no mutations (Table S5). For *Avr-Pi9*, amplicons were successfully generated for 48 isolates, whereas two isolates failed to amplify (Figure 5C). Among the 48 isolates, 28 were sequenced due to their virulence on K18 (carrying *Pi9*). No nucleotide variations were observed in the CDS regions of *Avr-Pi9* from these 28 isolates (Table S5).

## 4. Discussion

Rice blast, caused by the fungal pathogen *Magnaporthe oryzae*, poses a severe threat to global rice production and food security. The deployment of resistant rice varieties is widely recognized as the most efficient, environmentally friendly, and sustainable strategy for controlling this disease. However, the rapid genomic evolution of *M. oryzae*, driven by high genetic variability, presents a significant challenge to the long-term effectiveness of blast-resistant rice breeding programs [41]. Therefore, the continuous monitoring of the population dynamics and avirulence gene profiles of *M. oryzae* in key rice-growing regions is essential for guiding the rational breeding and deployment of blast-resistant rice cultivars. Jilin Province, located in northeastern China, is a major japonica rice-producing area, and the systematic characterization of the local *M. oryzae* population is crucial for safeguarding regional rice production.

In this study, we characterized the pathogenicity patterns, regional variations in virulence profiles, and inferred *Avr* compositions of *M. oryzae* in central Jilin Province, a core rice-growing region. A total of 294 field isolates of *M. oryzae* were collected from central Jilin Province in 2022, and their pathogenicity was evaluated using a set of rice MLs carrying different resistance genes. The results showed significant variations in pathogenicity among the tested isolates across different MLs. Specifically, 29.25%, 36.39%, 29.93%, and 4.42% of the isolates exhibited extremely high, high, moderate, and low pathogenicity, respectively. Notably, isolate JL3 displayed the highest pathogenicity against all tested MLs, indicating that it possesses strong virulence and may pose a potential threat to local rice production. Collectively, these findings suggest substantial changes in the pathogenicity of the *M. oryzae* population in central Jilin Province, which may undermine the effectiveness of existing blast-resistant rice varieties and pose a considerable threat to future rice production in this region.

Phenotypic assessments of the *M. oryzae* isolates against rice MLs revealed that more than 50% of isolates exhibited an incompatible phenotype with MLs carrying the *R* genes *Pi3*, *Pit*, *Pi7*, *Pikh*, *Pik*, and *Pia*. This indicates that these *R* genes represent effective resistance sources for breeding blast-resistant rice cultivars tailored to central Jilin Province. Furthermore, the effectiveness of these *R* genes varied among different cities in the study region: *Pit* showed the highest effectiveness in Changchun and Jilin cities, *Pi3* performed the best in Tonghua, and *Pikm* was the most effective in Liaoyuan. These regional differences in *R* gene effectiveness highlight the importance of regionalized resistance gene deployment based on local *M. oryzae* population characteristics. Noteworthily, all resistance assessments in this study relied on wound inoculation using detached leaves. Although this laboratory bioassay is efficient and repeatable, it cannot fully represent the resistance performance of intact plants or actual field conditions. Consequently, the practical application of these resistance genes should be considered cautiously. Further whole-plant inoculation and field verification are needed to confirm their durable resistance in natural rice production environments.

Our findings are partially consistent with those of a previous study by Wang et al., who collected 206 *M. oryzae* isolates from three japonica rice-growing regions in Jilin Province between 2019 and 2021 [42]. In their study, 81 isolates collected from the central semi-humid region of Jilin Province exhibited high virulence frequencies towards MLs carrying *Pish*, *Pi-ta*, and *Pi-ta2*, which aligns with our findings. However, a discrepancy was observed in the virulence frequencies toward *Pia* and *Pii*: Wang et al. reported high virulence frequencies of *M. oryzae* isolates against these two *R* genes, whereas our results showed only moderate virulence. This discrepancy is likely attributed to differences in the collection years of the isolates, as the genetic structure and virulence of *M. oryzae* populations can change dynamically over time. Additionally, the effectiveness of *R* genes varies across different geographic regions: for example, *Pikm* showed the highest resistance frequency in Jiangxi Province [43], while both *Pi9* and *Pikm* conferred resistance to more than 75% of the tested isolates in Hunan Province [6]. In contrast, our study demonstrated that *Pi3* was highly effective against local *M. oryzae* isolates in Jilin Province, emphasizing the regional specificity of *R* gene effectiveness and the need for region-specific resistance breeding.

Based on the gene-for-gene hypothesis, the functional *Avr* gene genotype of each *M. oryzae* isolate was inferred from its phenotypic performance on the corresponding MLs. The results showed that *Avr-Pi3*, *Avr-Pit*, and *Avr-Pi7* were the predominant *Avr* genes in the local *M. oryzae* population, with frequencies of 61.90%, 59.52%, and 56.80%, respectively. This is consistent with the dynamic balance between rice *R* genes and *M. oryzae Avr* genes [43]. Notably, *Avr-Pi11*, *Avr-Pita*, and *Avr-Pi9* displayed the lowest postulated frequencies in field isolates from Changchun, Jilin, Tonghua, and Liaoyuan, respectively. This suggests that the corresponding *R* genes (*Pi11*, *Pi-ta*, and *Pi9*) may not need to be prioritized in the current rice variety deployment in these regions, as the low frequency of their cognate *Avr* genes indicates that the local *M. oryzae* population has already evolved to overcome these *R* genes. Noteworthily, the *Avr* gene profiles inferred in this study have certain limitations. The phenotypic inference of *Avr* genes may be interfered by various factors, including unknown *R* genes in the MLs, virulence redundancy in *M. oryzae*, alternative immune pathways in rice, *Avr* gene silencing, or regulatory mutations in *M. oryzae*. These factors may lead to the inconsistent or false-positive predictions of *Avr* gene genotypes. Therefore, further molecular detection and functional validation are necessary to verify the inferred *Avr* gene profiles in future research.

Currently, the number of cloned rice *R* genes far exceeds that of identified *Avr* genes in *M. oryzae*, which limits our understanding of the molecular mechanisms underlying the rice–*M. oryzae* interaction. A key characteristic of *Avr* genes is their high genetic variability, which includes gene deletion, nucleotide substitution, and transposon insertion. These genetic variations enable *M. oryzae* to evolve virulent isolates that can overcome rice *R* genes, thereby reducing the durability of blast resistance [44,45]. For example, in Hunan Province, the *Avr-Pita* gene exhibits abundant single-nucleotide polymorphisms (SNPs) among different *M. oryzae* isolates, indicating extensive genetic variations [6]. In this study, three *Avr* genes (*Avr-Pizt*, *Avr-Pita2*, and *Avr-Pi9*) were amplified from 50 randomly selected isolates, and pathogenicity was assessed in the corresponding isolates. PCR products from isolates that were virulent to the corresponding MLs were subjected to sequencing, and the results revealed one SNP mutation in both *Avr-Pizt* and *Avr-Pita2*. These genetic variations may contribute to the high virulence frequencies observed against MLs carrying *Pizt* and *Pi-ta2*, respectively, as SNPs may alter the structure and function of *Avr* proteins, thereby preventing recognition by the corresponding R proteins.

Additionally, we observed that 18 isolates harboring *Avr-Pizt*, 17 isolates harboring *Avr-Pita2*, and 28 isolates carrying *Avr-Pi9* were still virulent to the corresponding MLs. We speculate that this phenomenon may be attributed to the suppressed expression or non-expression of the corresponding *Avr* genes. To verify this hypothesis, further studies are needed to investigate whether mutations exist in the promoter regions of *Avr-Pizt*, *Avr-Pita2*, *Avr-Pi9*, and other *Avr* genes, as promoter mutations can affect gene transcription. Moreover, the potential involvement of epigenetic modifications (e.g., histone acetylation/deacetylation and DNA methylation) in regulating *Avr* gene expression requires further validation by additional sequencing [6], and reverse transcriptional-PCR (RT-PCR) should be performed to quantify the expression levels of these *Avr* genes. Previous studies have demonstrated that transposon insertion is an important mechanism underlying *Avr* gene inactivation and virulence evolution in *M. oryzae*. For instance, a complete Pot3 transposon was detected within the coding sequence of *Avr-Pita* in the virulent isolate B2; this insertion was shown to transform avirulent strains into virulent ones, representing a unique molecular pathway through which *M. oryzae* can overcome the *Pi-ta* resistance gene [46]. Similarly, in Yunnan Province, China, some new virulent *M. oryzae* isolates have emerged that overcome the *Piz-t* gene with the solo-LTR insertion of the retrotransposon inago2 in the *Avr-Pizt* promoter region, frameshift mutations, and structural variations in *Avr-Pizt* [31]. In addition, *Avr-Pita* was successfully amplified from isolate 60/1-5, which was still virulent in *Pi-ta*-carrying cultivars. The authors speculated that a different mechanism may be involved in overcoming *Pi-ta*-mediated resistance, and future sequence analysis of *Avr-Pita* promoter region in isolate 60/1-5 will help determine whether transposon insertion or other genetic changes in the promoter region are responsible for this virulence [46]. These findings collectively indicate that multiple molecular mechanisms drive the virulence evolution of *M. oryzae*, and further investigation of these mechanisms is crucial for understanding the coevolution of rice and *M. oryzae*.

## 5. Conclusions

The continuous monitoring of *M. oryzae* pathogenicity patterns, regional variations in virulence profiles, and inferred *Avr* compositions is crucial for rice blast resistance breeding. This study systematically analyzed 294 isolates from central Jilin Province, uncovered key genetic shifts in the local pathogen population, and provided valuable insights for sustainable rice blast management. Highly pathogenic isolates dominated the local *M. oryzae* population, with significant regional variations in virulence compositions. The *R* genes *Pit*, *Pi3*, and *Pikm* were the most effective in Changchun/Jilin, Tonghua, and Liaoyuan, respectively. Isolates showed the highest virulence to *Pish* and *Pi11* MLs and the lowest to *Pi3* lines. The *Avr* genes *Avr-Pi3*, *Avr-Pit*, and *Avr-Pi7* were inferred in over 56% of the isolates. Thus, we propose a targeted breeding strategy emphasizing *Pit*, *Pi3*, and *Pikm* pyramiding in Changchun/Jilin, Tonghua, and Liaoyuan, respectively. Combined with long-term pathogen population surveillance, this strategy will facilitate the breeding of durably resistant rice varieties and stable rice blast control in Jilin Province.

## Figures and Tables

**Table 1:** Origins of field isolates used in this study.

Location	Isolate Codes	Total Isolates
CC	CC1–CC28, CC30–CC47, CC49–CC73	71
JL	JL1–JL4, JL8, JL9, JL11–JL18, JL20, JL22, JL23, JL25–JL30, JL32–JL38, JL40–JL73	64
TH	TH1–TH93	93
LY	LY1–LY66	66

## Data Availability

The original contributions presented in this study are included in the article/Supplementary Material. Further inquiries can be directed to the corresponding authors.

## Supplementary Materials

The following supporting information can be downloaded at: https://www.mdpi.com/article/10.3390/microorganisms14051017/s1, Figure S1: Distribution of sites in Jilin Province where *M. oryzae* samples used in this study were collected from the fields; Table S1: MLs with different resistance genes; Table S2: Primers used in this study; Table S3: Reaction patterns of 294 isolates; Table S4: List of inferred *Avr* genes in 294 isolates; Table S5: Allelic variations in *M. oryzae* avirulence genes.
