# Supplementary material for: Pathogenicity Analysis and Molecular Characterization of Three Avr Genes in Magnaporthe oryzae Population from Central Jilin Province
Source: Microorganisms. 2026 Apr 30;14(5):1017. doi: 10.3390/microorganisms14051017 (PMC13209718; doi:10.3390/microorganisms14051017)
Supplement: Supplementary file 1 [file microorganisms-14-01017-s001.zip › Figure S1.pdf]

A

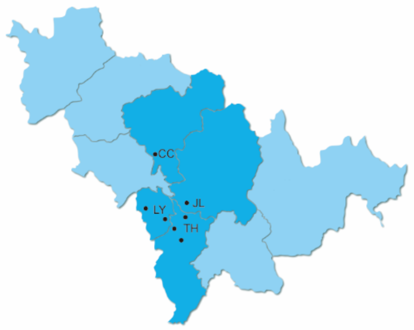

B

Location	Latitude and longitude
CC (Changchun)	N43°48′|E125°24′
JL (Jilin)	N42°56′|E125°58′
TH (Tonghua)	N42°41′|E125°48′
	N42°16′|E125°72′
	N42°38′|E126°17′
LY (Liaoyuan)	N42°19′|E125°12′
	N42°48′|E125°02′

Figure S1. Distribution of sites in Jilin Province where *M. oryzae* samples used in the study were collected from the fields.
